# Structural and Computational Biology in the Design of Immunogenic Vaccine Antigens

**DOI:** 10.1155/2015/156241

**Published:** 2015-10-07

**Authors:** Lassi Liljeroos, Enrico Malito, Ilaria Ferlenghi, Matthew James Bottomley

**Affiliations:** Novartis Vaccines & Diagnostics S.r.l. (a GSK Company), Via Fiorentina 1, 53100 Siena, Italy

## Abstract

Vaccination is historically one of the most important medical interventions for the prevention of infectious disease. Previously, vaccines were typically made of rather crude mixtures of inactivated or attenuated causative agents. However, over the last 10–20 years, several important technological and computational advances have enabled major progress in the discovery and design of potently immunogenic recombinant protein vaccine antigens. Here we discuss three key breakthrough approaches that have potentiated structural and computational vaccine design. Firstly, genomic sciences gave birth to the field of reverse vaccinology, which has enabled the rapid computational identification of potential vaccine antigens. Secondly, major advances in structural biology, experimental epitope mapping, and computational epitope prediction have yielded molecular insights into the immunogenic determinants defining protective antigens, enabling their rational optimization. Thirdly, and most recently, computational approaches have been used to convert this wealth of structural and immunological information into the design of improved vaccine antigens. This review aims to illustrate the growing power of combining sequencing, structural and computational approaches, and we discuss how this may drive the design of novel immunogens suitable for future vaccines urgently needed to increase the global prevention of infectious disease.

## 1. Introduction

Vaccines are one of the most successful medical interventions in human history and estimated to prevent more than 2.5 million deaths every year [1, 2]. In essence, vaccination is about convincing the immune system to treat a noninfectious artificially introduced substance as an invading pathogen and to raise an immune response that would protect the vaccine from future infection. Vaccination ideally induces an immune response equal to or better than that caused by natural infection. As a result, long-term immunity against a pathogen can be obtained that prevents the individual from disease as well as from transmitting the pathogen thus contributing to the herd protection of the whole society. The history of vaccination is considered to have started with Edward Jenner's experiments in 1796 showing that vaccination with pus from milk maids' blisters caused by cowpox protected humans against smallpox. Since then, the science of vaccines has come a long way, from using inactivated pathogens or toxins and attenuated live pathogens to recombinant subunit and glycoconjugate vaccines, and most recently towards structurally designed epitope-focused vaccines [3].

In its simplest form, effective vaccination can be achieved with inactivated or attenuated pathogens. This has been and still remains the best available solution against many diseases such as measles, mumps, and varicella. Such vaccines have resulted in complete or almost complete eradication of devastating diseases like smallpox and polio. Despite the proven effectiveness in many cases, inactivated pathogens do not always generate adequate protection and attenuated pathogens have safety concerns caused by possible reverse mutations. Further, the logistics of immunizing people in developing countries with live attenuated vaccines is problematic due to the often strict requirements of an uninterrupted cold chain to keep the pathogens alive. Live attenuated vaccines also pose an increased risk for immunocompromised subjects that may not be able to respond adequately to limit the infection.

In the 1970s, glycoconjugate and recombinant subunit vaccines revolutionized the field allowing the development of safer and more effective vaccines. Glycoconjugate vaccines superseded the previous capsular polysaccharide vaccines, enabling efficient T-cell activation required for long-term immunity. The general mechanism of how the carrier protein helps in T-cell engagement is still, however, unconfirmed [4, 5]. Glycoconjugate vaccines have proven successful against a number of bacterial pathogens such as* Haemophilus influenzae* type B,* Neisseria meningitidis* serotypes A, C, W-135, and Y, and* Streptococcus pneumoniae* [6]. All of the current glycoconjugate vaccines target bacterial pathogens, but the technology is also potentially suitable against viruses like HIV, since their main antigens are highly glycosylated and some of the broadly neutralizing antibodies (bnAbs) have been shown to bind to glycan moieties on the envelope attachment and fusion protein (Env) surface [7].

The advent of recombinant DNA technology in the late 1970s was quickly adopted in the vaccines field. The new techniques enabled heterologous large-scale production of single proteins from pathogens and their modification in order to optimize proteins for vaccine use (e.g., by detoxification of undesirable catalytic activity). Recombinant subunit vaccines initially proved their usefulness against viruses like hepatitis B virus [8, 9] and human papilloma viruses [10] but have since also been used in bacterial vaccines. An example of this is the recently approved 4-component vaccine against* Neisseria meningitidis* serogroup B (MenB) (Bexsero) that is composed of three recombinant proteins and an outer membrane vesicle preparation from the bacteria. This MenB vaccine is also the first vaccine approved for human use for which the starting point of development relied on genomic data and bioinformatics to select the initial pool of antigen candidates by reverse vaccinology (RV) [11].

**Figure 1:**
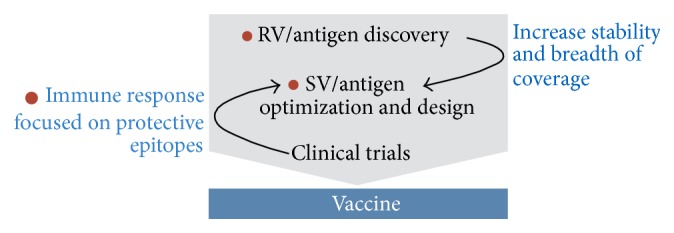
A simplified pipeline for a vaccine development project. Contributions from modern, mainly SV approaches are indicated in blue, while red dots indicate steps aided or made possible by computational methods.

The great developments in the speed of DNA sequencing and the associated computational methods have enabled large-scale antigen mining by RV and it has already been used for several pathogens [12–17], mainly bacteria, but recently also for herpes simplex virus [18] to find surface expressed or secreted antigen candidates. Initially, candidates are often found to be suboptimal in terms of stability, safety, immunogenicity, or generating broad protection against all strains of the pathogen. Structural vaccinology (SV) is a rational approach that can be used to address these issues. Major aims in SV are the identification of protective B-cell epitopes on the antigens and optimizing the antigens in terms of stability, epitope presentation, ease of production and safety. SV is a symbiosis between experimental methods like X-ray crystallography, electron microscopy and mass spectrometry, and computational methods like structural modeling, computational scaffold design and epitope prediction. Recent breakthrough examples in the fields of respiratory syncytial virus (RSV) [19], human immunodeficiency virus 1 (HIV-1) [20], MenB [21] and group B streptococcus (GBS) [22] indicate that SV has the potential to become another revolutionary step in vaccine development given that many of the important infectious diseases currently not preventable by vaccines are not amenable to traditional approaches. In this review we discuss the experimental and computational aspects of three key modules of a modern vaccine development pipeline: reverse vaccinology, epitope characterization, and structure-based antigen optimization and design. Of the myriad of experimental methods, algorithms, and software developed for these approaches, we highlight the ones we consider of highest practical relevance for vaccine development. We summarize our view of the whole vaccine development process for current and near future vaccines in Figure 1.

## 2. Genomic Era, Next-Generation Sequencing and Reverse Vaccinology

Traditionally, vaccine antigen candidates for subunit vaccines have been selected based on experimental data on function, abundance, and immunogenicity. These methods may have overlooked many potentially excellent candidates present with high abundance under the natural conditions during colonization and infection but which may have been present only in lower amounts on the pathogen surface during experimental characterization of antigen expression under laboratory conditions [23]. Initially with shotgun sequencing approach [24] and more recently with next-generation sequencing [25] allowing rapid determination of sequences of whole genomes, vaccine candidate discovery especially on bacterial pathogens has shifted more towards computational prediction of suitable vaccine antigen candidates from genomic sequences using RV. To date, a number of different methodologies have been developed for high-throughput genomic sequencing of which the most commonly used are the sequencing by synthesis (Illumina), ion semiconductor detection (Life Technologies), pyrosequencing (Roche Diagnostics), and more recently single-molecule real time sequencing (PacBio) [26]. Common to most NGS technologies, the output is a set of millions or even billions of short (50–700 bases) sequence reads accompanied by a base-call quality metric. Thus, a major part of any NGS project is assembling the short reads together in a precise and reliable manner. Assembling raw sequencing data of a whole genome is still not trivial and every sequencing technology has its typical types of reads characteristics and sequencing errors that have to be accounted for. A detailed review of assembly methods is beyond the scope of this paper, but we suggest looking into other reviews for a description of the current state of the art [27, 28].

With the number of sequenced bacterial genomes already in the tens of thousands and availability of multiple, for some bacteria >100, complete genome sequences, it has become possible to use a core set of genes shared by all strains in the RV computational analysis [16, 38]. Alternatively, the availability of multiple genome sequences from one species enables a comparison between the genomes of pathogenic and nonpathogenic strains, which can reveal genes important for pathogenesis that can often be good vaccine antigen candidates [39]. The first step of any RV approach is to predict all ORFs from the genomic sequence and pass them individually through several computational selection filters. In the classical RV approach the features selected are based on the assumption that, in order to be available for interactions with protective antibodies, the antigen has to be either surface-associated or secreted [11]. Features that are typically computationally analyzed include transmembrane domains, leader peptides, homology to known surface proteins, lipoprotein signatures, outer membrane anchoring motifs, and host cell binding motifs such as RGD. The main software that was used in the first RV projects and has retained its popularity is PSORT, now in its third generation for prokaryotic sequences [34]. Since the initial version of PSORT, a number of other software packages for protein cellular localization prediction in bacteria have been published, most using support vector machines on experimental datasets to train the software for prediction [40–44].

Since PSORT remains one of the most widely used subcellular localization prediction software packages in RV, we briefly describe its underlying principles here. PSORT comes with its own database PSORTdb [45] composed of several thousand proteins with experimentally verified subcellular localization that it uses as a reference set for queries. PSORT is a modular program analyzing several features known to be relevant for protein localization like sequence homology to proteins with known localization, signal peptides, amino acid composition, and motifs. In its latest versions PSORTb 2.0 and PSORTb 3.0 the software has also been trained against an extended PSORTdb dataset using an* n*-peptide composition-based support vector machine, a kernel learning algorithm to improve the percentage of proteins for which a prediction is reported. PSORTb 3.0 covers all prokaryotes, including archea and bacteria with atypical cell wall or membrane topologies, and was reported to predict the subcellular localization with over 95% precision and with a recall of over 90% for both Gram negative and positive test datasets [34].

In general, based on subcellular location only, a large fraction (typically around 30%) of the whole proteome gets selected. In the first applications of RV on MenB an astonishing number of 350 candidates and for GBS 312 candidates were expressed and tested in mice to find promising vaccine antigens [11, 16]. Carrying such a large panel of proteins through the whole workflow of cloning, expression, and purification and above all animal experiments is impractical, which has led to the development of narrowing methods combining computational and experimental, mainly mass spectrometric (MS), methods [46–48]. Identification of an antigen candidate by MS provides proof of expression and can confirm surface localization. Expression can, however, vary in different culture conditions and what is observed* in vitro* may not always be representative of* in vivo* conditions. Several purely computational methods, summarized in Table 1, have also been specifically developed for RV purposes. NERVE added, on top of PSORT subcellular localization analysis, exclusion of multipass membrane proteins and human homologues and positive selection for adhesin-like features, that is, proteins likely to have host cell adhesion functions. Raising antibodies against adhesins can have an inhibitory effect on colonization and infection [33]. Vaxign further added MHC I and MHC II epitope prediction. Jenner-Predict, while using some of the same filtering criteria as NERVE and Vaxign, put more weight on the known host-pathogen interaction domains on proteins [32]. Vacceed framework extended the vaccine antigen prediction also to eukaryotes [35], which are typically much more challenging targets due to their complexity compared to prokaryotes. Also approaches based on existing experimental data on features of known protective antigens have been used in vaccine antigen prediction. VaxiJen uses an alignment-free approach and is based on statistical methods using auto-cross covariance transformation of protein sequences into uniform vectors of principal amino acid properties [37]. For whole proteomes, VaxiJen was, however, reported to identify a set of proteins almost as large as traditional RV approaches [49]. To increase selectivity, another method based on identification of structural and functional features in known bacterial protective antigens and using this data to discover new protective antigens was developed [49]. This method relies on databases and tools such as Pfam and SMART to find features correlated with protectivity of antigens and searches for these features within the protein coding sequences of a particular genome. The program is designed not to take into account any localization signals, enabling recognition of intracellular antigens, the majority of which are T-cell antigens. This “protectome analysis” method was reported to be more selective than VaxiJen leaving only a few dozen candidate antigens identified from a whole bacterial proteome to test experimentally, while still being able to select all the antigens in the MenB (Bexsero) and* Bordetella pertussis* (Daptacel) vaccines.

Once a panel of candidate antigens has been selected, they need to be tested in preclinical animal models. The challenge here is that for many pathogens the correlates of protection are not clear. In other words, the animal experiments may not be reliable indicators of protection in humans, and following wrong or too few metrics, like immunogenicity only, can misguide antigen selection. Nevertheless, the candidate antigens have to fulfill at least three general key features: they have to be immunogenic, they have to be conserved and expressed in natural infection, and they have to be safely tolerated. Since the majority of vaccine-induced protection is generally based on antibodies, the antigen candidate has to elicit measurable, preferentially high antibody titers. Also, if a protein is toxic to experimental animals, the risks of use in humans are too high to consider it as a vaccine antigen as such. In some cases, like that of MenB, at the time of antigen selection it was known that, for protection, antibodies that have complement-mediated bactericidal activity are required, which simplified the selection process [50].

With many viruses, there are few antigen candidates to choose from and reverse vaccinology is not required. However, surface-exposed viral antigens often exhibit high degrees of sequence variability and conformational heterogeneity, which can make it difficult to produce stable immunogenic conformations and find epitopes conserved across different strains of the viruses. Independently of how one arrives at the selected antigen(s), the next step in understanding the molecular basis of protection and optimizing the antigen is to determine its structure and find the epitopes where protective antibodies bind.

## 3. Structural Characterization of Antigens and Antigen-Antibody Interactions

From the initial 7 entries deposited in the Protein Data Bank (PDB) when it was established in 1971, the total number of macromolecular structures deposited is currently >100,000, a vast collection of data achieved due to many technological innovations and advances in experimental methods of structure determination. The structures of most vaccine antigens and their epitopes can nowadays be determined and used for advanced, rational, structure-based vaccine design [51]. Knowing the structures of antigens that are candidates to become vaccines enables rational design to fine tune their presentation to the immune system or to facilitate their manufacturing. At the same time, structures of antigen-antibody complexes provide useful information to understand the molecular nature of host-pathogen interactions and of pathogen- or vaccine-induced antibody responses. This information can in turn help to elucidate the effects of immunization and also provides useful knowledge for the development of general principles and computational tools for* in silico* predictions of protective epitopes [52]. Systematic structure-based approaches applied to vaccine research can potentially save time and resources and can also aid the development of vaccines for difficult antigen targets that resist other traditional approaches [53].

### 3.1. Epitope Mapping and Discovery

Knowing the exact regions of an antigen that are recognized and bound by antibodies provides essential information for antigen engineering and can be used to guide vaccine design and optimization. The experimental methods necessary to obtain such information are collectively called “epitope mapping,” and their important role in the early stages of vaccine design has been recognized for several years [54]. Importantly, the postgenomic era and the rapidly increasing number of available structures of antigens and antigen-antibody complexes now open new possibilities for the development of novel tools that can reliably predict protective epitopes. Using either the sequence or the structure of antigens that are target vaccine candidates, these methods could drastically reduce experimental efforts in discovering epitopes needed for the design of vaccines. Below, we will first review recent applications of epitope mapping methods with a focus on the high-resolution mapping by X-ray crystallography and the emerging potential of cryo-EM, and we will then review the current status of computational methods for the prediction and design of B-cell epitopes. Reviews of computational methods for the prediction of T-cell epitopes have been provided previously [55, 56].

#### 3.1.1. Experimental Epitope Mapping

An epitope can be defined as the collection of atoms that directly contact or bind an antibody. Electrostatic attractions, water-mediated hydrogen-bond networks, and long-range forces such as ionic and hydrophobic interactions contribute to the overall binding affinity between an antigen and an antibody [57–60]. In addition, it has been recently suggested that allosteric effects between constant and variable regions of antibodies play a role on antigen affinity or specificity [58, 61]. B-cell epitopes can be either linear (continuous or sequential) or conformational (discontinuous). While linear epitopes are short peptides made of a contiguous amino acid sequence fragment of a protein, conformational epitopes are composed of noncontiguous amino acids (in primary sequence) that are brought into close proximity within the folded 3-dimensional protein structure. It is estimated that most B-cell epitopes (up to 90%) are conformational [62].

An empirical approach to epitope mapping is still essential in order to generate reliable information about antibody-antigen interactions, and many experimental methods of epitope mapping are available. Among those methods that do not require knowledge of the tertiary structure, of either the antigen or the antibody, are (i) the use of synthetic libraries of peptide fragments to scan their binding by an antibody (Pepscan); (ii) the use of bacteriophages (or other organisms) displaying libraries of peptides on their surface to study their binding by antibodies (Phage Display); (iii) various strategies of mass spectrometry (MS) such as epitope extraction, excision, differential chemical modification, and more recently hydrogen-deuterium exchange (H/DX-MS); (iv) solution NMR epitope mapping [63, 64]. In contrast with the information provided by the mainly sequence-based methods cited above, X-ray crystallography delivers a clear visual definition and an atomic-level description of the epitope and paratope atoms forming the antigen-antibody interface. To date there are over 100 nonredundant antibody-antigen (i.e., Fab-protein) complex structures deposited in the PDB, a number that is likely to grow further due to the increasing ease in obtaining and producing human Fabs [65].

An example of epitope mapping approaches has recently been published, showing the importance of employing different methods when a high-resolution picture of the epitope-paratope interface is not available [66]. After failing to obtain crystals of the complex between the programmed death ligand 1 protein (PD-L1) and a monoclonal antibody that targets PD-L1 binding to its receptor, the programmed death protein 1 (PD-1), Hao et al. used limited proteolysis, HDX-MS, mutational studies, and surface plasmon resonance (SPR) to reveal and characterize the epitope.

Another example of interdisciplinary approaches to epitope mapping that instead illustrates the limitations of some methods has been reported for the MenB factor H binding protein (fHbp) and its interaction with a monoclonal antibody (mAb 12C1) [67]. Here, the crystal structure of the complex fHbp-Fab 12C1 was solved at 1.8 Å resolution, revealing high-resolution details on an extensive epitope-paratope interface involving the variable heavy (VH) and variable light (VL) chains of mAb 12C1 and both the N- and C-terminal domains of fHbp. This interface involves 23 fHbp and 33 Fab residues and generates buried surfaces of ~1000 Å on fHbp and ~880 Å on Fab 12C1. In addition to the crystal structure of the complex, also HDX-MS revealed a large, discontinuous, conformational epitope, though with broader boundaries. Instead, Pepscan and Phage Display identified partial epitopes only and, as expected, of exclusively linear nature. However, these partially mapped and linear regions were also part of the main epitope as identified both by the crystal structure and by HDX-MS [67].

Epitope mapping by X-ray crystallography is probably one of the most powerful and important applications of structural biology in the field of vaccines research. But it is important to recognize that X-ray crystallography cannot be considered a universal solution to epitope mapping, as there are also limitations such as (i) the generation of crystals typically requiring large amounts of sample material, (ii) the lack of certainty that any protein, or antigen-antibody complex, will produce high-quality crystals, and (iii) the unpredictable timelines for the crystallization and structure determination processes that while in the most favourable cases can be very short (days-weeks) or indeed may even never be achieved. As an alternative, for small antigens (<30 kda), solution NMR can also be a rapid and accurate method for epitope mapping and is particularly useful if HSQC peak assignment for the antigen is already available. Examples of NMR epitope mapping include work on MenB and gonococcus fHbp and the DIII domain of dengue virus E protein [68–70].

An emerging method in macromolecular structure determination is cryoelectron microscopy (cryo-EM). Due to the impressive recent progress mainly in electron detectors and software algorithms, it is now possible to determine protein cryo-EM structures to quasiatomic resolution using single-particle methods in 3D reconstruction [71–74]. The great attraction of single-particle cryo-EM compared to X-ray crystallography is that it requires only micrograms of sample and crystallization is not required. Moreover, since images of individual molecules are obtained, computational classification methods can be used to reveal multiple conformational states. Obtaining high-resolution reconstructions (<4 Å) is however greatly facilitated by having a rigid, homogenous complex, preferably several hundreds of kilodaltons in molecular weight. Yet, even low-resolution EM maps can be useful in providing information on the overall architecture of a protein or a protein complex and intermediate-resolution EM maps can already offer insights into the arrangement of domains and localization of functional sites on the macromolecules. Docking of X-ray structures of individual subcomponents in EM maps can be done with high precision, thus increasing the apparent resolvability of the results and making it possible to extract atomic details from the maps. Flexible fitting methodologies can be used to further improve the fitting of X-ray structures into the EM density maps [75]. Recent examples, where X-ray structures have been used to help detailed interpretation of cryo-EM maps, include work on alternative function-related conformational states of a complex, a protein in complex with a cofactor and an antigen-antibody complex [76–78].

Several recent publications illustrate the usefulness of cryo-EM for epitope mapping. Aiyegbo and coworkers described a hybrid method approach for epitope mapping, based on single-particle cryo-EM and enhanced H/DX-MS to determine the location and mode of binding of RV6-25 Fab directed against the VP6 epitope of human* Rotavirus* [79]. Interestingly, the structure of the RV6-25 Fab attached to the double-layered particle (DLP) complex determined by cryo-EM indicated a rather complex binding pattern that revealed differences in accessibility of the VP6 epitope depending on its position in the type I, II, or III channels (located at the icosahedral 5-fold and 3-fold axes of which the former serve as egress points of nascent viral mRNA during viral transcription) (see Figure  2 in [79]). These variations in the accessibility of the RV VP6 capsid layer led to position-specific differences in occupancy for binding of the RV6-25 Fab. A second innovative publication by Bannwarth and collaborators described a new structural approach to characterize in 3D the poliovirus type I epitopes in virus-antibody immune complexes [80]. Briefly, the inactivated polio vaccine (IPV) contains serotypes 1, 2, and 3 of poliovirus. All three serotypes share the D-antigen, which induces protective antibodies. The antigenic structure of PVs is composed by at least four different antigenic sites; thus the D-antigen content results in the combined activity of multiple epitopes. Characterization of the epitopes recognized by the different mAbs was fundamental to map the entire virus surface and ensure the presence of epitopes able to induce neutralizing antibodies. In their new approach the authors describe how combination of single-particle cryo-EM with X-ray crystallographic data allowed the identification of the antigenic sites for these mAbs. The generation and comparison of five different 3D EM maps generated from five different specific Fab-virus and one mAb-virus complex allowed the identification of exposed amino acid residues and finally the mAb-antigen sites. This new approach can be used to map the whole “epitopic” viral surface and provide a comprehensive picture of main epitopes on the surface.

In addition to the examples described above, cryo-EM has successfully been used to map epitopes on several icosahedral viruses, generally difficult to crystallize due to their large size [81, 82]. Cryo-EM also allows characterization of antigen-antibody complexes* in situ*, for example, on enveloped virus surface when tomographic reconstruction methods are used. Such approaches have allowed characterization of influenza HA in complex with a stem-directed mAb showing that the stem is accessible for mAb binding even though the virion surface is densely packed with HA and NA spikes [83].

Fabs have also been used to increase the size of the protein of interest for cryo-EM imaging and reconstruction purposes [84]. Since small (<100 kDa) proteins have up to now been difficult to reconstruct due to lack of evident features and consequent failure in particle alignment, Fabs with their well-known overall structure can be used to aid alignment and validation. Not only does this enable the structure determination of the protein of interest, but simultaneously produce a structure of the antigen-antibody complex. Thus, antigen-Fab complexes are in fact easier to reconstruct than small antigens alone, which extends the capabilities of cryo-EM to studies of smaller antigens. In a recent example, a Fab from HIV bnAb PGV04 was used to help reconstructing an Env-Fab complex to 5.8 Å resolution [77].

Although X-ray crystallography is still the leading method for high-resolution antigen-antibody interaction characterization, single-particle cryo-EM holds the promise to become complementary in cases where crystallography is not possible or feasible. Furthermore, since cryo-EM can deal with heterogeneous samples, it may in the future become possible to characterize multiple complexes (e.g., from polyclonal sera) within the same sample, an important aspect for throughput and completeness in characterizing the full repertoire of antigen-antibody interactions, rather than just a few mAb-antigen interactions.

#### 3.1.2. Computational Methods for Epitope Prediction and Design

Epitope mapping studies performed over the last few decades have provided a wealth of information on epitope-paratope interfaces that have allowed a certain sophistication in the definition of an epitope [85]. Some common themes of antigen-antibody interactions are starting to emerge, with potential benefits for the development of computational methods for the reliable identification of B-cell epitopes. Also, the constant evolution and refinement of computational methods for protein folding and design, driven by the growth of available protein structures in the PDB, may now further aid in elucidating the molecular bases of antigen-antibody interactions.

Starting in the early 1980s, several sequence- and structure-based B-cell epitope prediction methods have been developed, as extensively reviewed elsewhere [52, 86]. However, developing robust computational methods for epitope prediction has proven to be a very difficult task, and their predictive performance remains far from ideal. Among the possible reasons for the limitations of current epitope prediction tools are the belief that we still possess somewhat weak or wrong hypotheses on the true nature of B-cell epitopes and on the structural bases for the ability of protective antigens to elicit functional antibodies [57, 87]. For example, the evidence that any residue of an antigen may become an epitope under certain circumstances poses a serious complication for most of the prediction methods [88]. However, most of the currently available prediction algorithms try to discriminate between epitopic and nonepitopic antigen surface residues [58]. Another obstacle to obtaining reliable predictions is the lack of large robust benchmark datasets and standard data formats, for which the community has proposed several solutions [88]. In support of the need of more robust benchmarks, the inclusion of additional biological information has been shown to greatly enhance prediction performances [89].

The most common computational methods of epitope prediction are sequence-based, and they specialize on predicting linear or continuous B-cell epitopes. These methods initially utilized sequence profiling by use of amino acid scales, where hydrophobicity, flexibility, solvent accessibility, or other physicochemical properties scales assign a propensity value to each amino acid, thus measuring their tendency to be part of a B-cell epitope [90]. These methods were later exhaustively reviewed and questioned, showing how almost 500 propensity scales performed only slightly better than random and thus leading to the conclusion that they cannot yet be used to predict epitope location reliably [91]. The use of machine-learning, knowledge-based methods was subsequently introduced in order to increase accuracy and reliability of the predictions.

Several lines of evidence indicate that most epitopes are conformational [62], and prediction of discontinuous or conformational epitopes presents further challenges as they require as a prerequisite the antigen structure. Indeed, all discontinuous epitope prediction methods currently available require the 3D structures of the antigen, which may in some cases be very difficult or impossible to obtain [92]. Despite continuous incremental advances in computational tools for the* in silico* prediction of 3D protein structures [93], the prediction of discontinuous epitopes still preferentially requires the experimental 3D structure to increase reliability. Some of the earlier approaches to conformational epitopes prediction used correlations of known epitopes with crystallographic temperature factors, protrusions from the protein globular surface, solvent accessibility, and flexibility. Also, both protein-protein binding site prediction tools [94] and docking algorithms [95] were introduced and tested for epitope prediction.

It has been recently estimated that the accuracy of continuous B-cell epitope predictions methods can reach 60–66% [52]. Importantly, recent computational and experimental validation of both continuous and discontinuous epitope predictions made with the most well established methods currently available show that they all still perform rather poorly [96].

More recent developments of computational methods for epitope prediction include the methods of electrostatic desolvation profiles (EDP) [97] and matrix of local coupling energies (MLCE) [98–100]. Both of these methods aim to elucidate the physicochemical determinants of antibody recognition by an antigen, starting from the structure of the antigen and the* in silico* analyses of its surface properties. This in turn allows making hypotheses on the optimal interface formation for protein-protein complexes in general (EDP) and for antigen-antibody complexes (MLCE). In particular, the MLCE algorithm takes into account both dynamic and energetic properties of a protein surface, looking for sites that because of their intrinsic low-intensity energetic couplings with the rest of the protein will likely undergo conformational changes, as well as mutations with minimal energetic expense, which are both desirable properties for antigenic epitopes and will influence the way an antigen-antibody complex forms. The MLCE does not require previous knowledge on antibody binding or the structure of an antigen-antibody complex, and as such it can be applied to any isolated protein antigen. Once those regions that are minimally coupled to the rest of the protein or the antigen, thus likely involved in antibody recognition, are localized, it is possible to introduce mutations that will increase the affinity for the antibody and at the same time will not affect the antigen's overall stability. Or it is possible to engineer stable regions of the antigen as more stable or dominant conformation of the one found in the original structural background of the entire antigen. Successful applications of the EDP and MLCE methods have been recently reported, which helped elucidate antigenic regions or immunogenic epitopes of several vaccine target candidates from* Burkholderia pseudomallei* (namely, BPSL1050 [101], the oligopeptide-binding protein A (OppABp) [102], the flagellar hook-associated protein (FlgKBp) [103], and the acute phase antigen BPSL2765 [104]).

A novel antibody-dependent prediction method has been recently introduced that instead of classifying Ag residues a priori as epitopic or nonepitopic will predict the potential match between a given Ab and a given epitope [105]. In addition to the antigen structure, this method also utilizes antibody sequences or structures and thus it promises to bring a new paradigm in epitope prediction by trying to predict which region of an antigen will bind a specific antibody or group of related antibodies, rather than any generic antibody. This concept is similar to the one used for T-cell epitopes prediction and specifically in the assumption of the specific major histocompatibility complex molecule presenting the epitope, rather than the antigen [55]. Combined with the growing field of immunoglobulin repertoire sequencing, this new approach promises to significantly increase the accuracy of B-cell epitope prediction methods. Also, by using antibodies structural or sequence information, this method might help focus the search for epitopes on certain antigenic regions and thus overcome the limitation of the antigen surface as a potentially continuous landscape of epitopes [58]. For example, groups of clonally related antibodies will often bind to the same, or similar, antigenic sites. By determining the high-resolution structures of these antibody-antigen complexes and by analyzing their interaction, a clearer picture of the molecular bases for recognition and binding may emerge, to be then exploited to improve prediction performances [106]. A recent study on the D8 protein of the vaccinia virus (VACV), which is a target of neutralizing Abs elicited by the smallpox vaccine, shows how this computational prediction method performs better than the state-of-the-art prediction methods available [107]. Importantly, in this same study it was shown how a significant increase of the prediction performance can be obtained when combining the antibody-specific predictions with relevant experimental data.

The sections above have described how novel candidate antigens can be discovered initially by sequencing and analyzing the genome of a pathogen and how structural biology—enriched by the combined addition of immunological and epitope mapping data—can yield highly detailed information on the most immunogenic and protective regions of such antigens. The following sections aim to illustrate how this information can drive the design of improved vaccine antigens. In general, antigen optimization strategies can be divided into two main branches, one branch aiming for better antigenic properties in terms of presentation of epitopes that elicit neutralizing or protective antibodies broadly reactive against antigens from multiple strains of the target pathogen; the other branch—equally important—aiming for structural stabilization, homogeneity, ease of production, and safety of the antigen.

## 4. Overcoming the Challenge of Antigen Sequence Diversity

Many pathogens manage to escape the host immune system by encoding surface-exposed antigens that exhibit high amino acid sequence diversity. The extent of this diversity can range considerably, depending on the pathogen. The “changing face” presented by a variable pathogen represents a challenge for the host immune system and consequently for the successful design of vaccines with broad coverage. One example where this issue has been tackled, with promising preclinical results, regards the antigen fHbp of* Neisseria meningitidis*.

FHbp is a highly immunogenic 28 kDa lipoprotein present on the surface of the majority of meningococcal strains. It was identified both by the computational reverse vaccinology strategy and by more traditional membrane-fractionation methods and is an effective antigen included in two recently licensed vaccines that protect against MenB (Bexsero, Trumenba), as reviewed recently [108]. There are now over 800 unique fHbp peptide sequences deposited in the public* Neisseria* database (http://pubmlst.org/neisseria/fHbp/) [109], hundreds of which are from MenB strains. The latter has implications when considering the design of a protein-based vaccine against MenB; that is, in contrast with the highly successful glycoconjugate vaccines that efficiently target the capsular polysaccharides of serogroup MenA, C, W, and Y [110], a protein-based vaccine should elicit diverse cross-reactive antibodies affording coverage of as many strains as possible, where protein antigens may display extreme sequence diversity. Computational and immunological analyses of these fHbp proteins allowed their grouping into three major sequence variants, which have as little as ~65% sequence identity between variant groups (but typically >90% identity within variant groups) which explains why the different variants are immunologically distinct [111]. In short, it appears that each meningococcal strain can use one of three immunologically different fHbp molecules in order to achieve factor H binding and thus downregulation of the host alternative complement pathway to promote its survival in the blood [112]. This antigenic variability promotes evasion of anti-fHbp directed immune responses, because the different fHbps are not broadly recognized by the antibodies previously induced by antigens of other variant groups.

**Figure 2:**
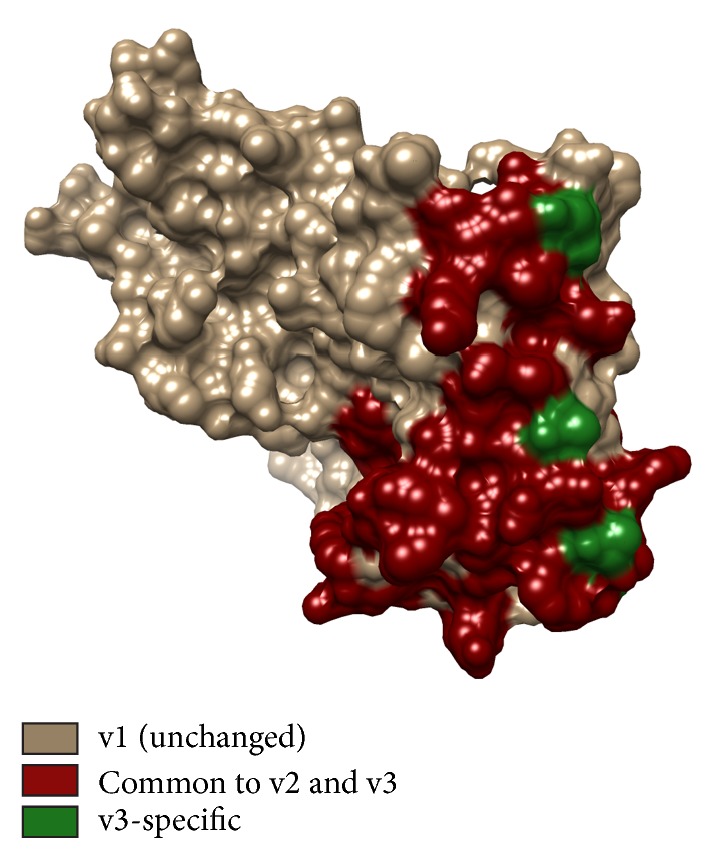
Broad coverage chimeric fHbp generated by rational design as described in [21]. The surface of variant 1 fHbp used as a scaffold is shown in brown. The engineered area carrying heterologous epitopes is colored in dark red (residues in common between variant 2 (V2) and variant 3 (V3)) and in green (variant 3-specific residues).

In order to overcome the meningococcal fHbp antigen sequence diversity, attempts were made to generate a single fHbp antigen capable of eliciting cross-reactive antibodies sufficient to enable bactericidal activity against all meningococcal strains. The three-dimensional (3D) structures of fHbp determined by NMR spectroscopy and later also by X-ray crystallography revealed a molecule composed of two similarly sized domains: an N-terminal taco-shaped beta-barrel and a C-terminal beta-barrel [113–115]. From this structural starting point, Scarselli et al. designed chimeric fHbp molecules, using variant 1.1 fHbp as a scaffold to display surface patches representing epitopes from fHbp variants 2 and 3 (Figure 2) [21]. The size and location of the grafted surface patches were selected and designed on the basis of two key analyses. Firstly, computational analyses of nonredundant antibody-antigen complex structures indicated that the typical epitope-paratope interface involves from 600 to 2000 Å^2^ surface area on each molecule [85, 116], thus suggesting the need to genetically engineer relatively large new surface patches. Indeed, single amino acids grafted from v2 or v3 were insufficient to elicit cross-reactive antigenicity. Secondly, epitope mapping studies performed using sera obtained from mice immunized with fHbp molecules of each variant group (v1, v2, and v3) suggested that the most immunogenic and protective epitopes of v1, v2, and v3 lay in nonoverlapping regions of the structure—with the C-terminal domain of fHbp harboring most of the v2 and v3 epitopes. Thus, combined sequence- and structure-based inputs led to the computational design of numerous partially overlapping patches each containing a sufficient number of new surface-exposed residues that could potentially form at least one v2- and/or v3-specific conformational epitope on the v1 scaffold. Over 50 mutants were prepared and used to immunize mice. The resulting sera were tested in serum bactericidal assays for their ability to induce complement-mediated killing of MenB strains displaying fHbp molecules of v1, v2, or v3 sequence types. This approach led to the successful design of a novel antigen able to induce broadly protective bactericidal responses against MenB [21].

A somewhat similar approach was recently used by Rippa et al. to graft fHbp v1-specific epitopes onto the gonococcal orthologue of fHbp (Ghfp) [68], which was previously reported to induce strongly bactericidal antibodies against fHbp v2 and v3, but only to a limited extent against fHbp v1 strains of MenB [117]. Immunization of mice with this Ghfp scaffold chimera displaying v1 epitopes was shown to induce bactericidal antibodies against all three fHbp variants, thus demonstrating that combining epitopes from different yet closely related species can be a viable strategy to generate broadly protective antigens [68]. To our knowledge, these two fHbp-centric studies currently represent the only demonstration that the antigenic sequence diversity of a pathogen can be overcome by computational and structure-based design. Clearly, a prerequisite and potential obstacle is detailed knowledge of the 3D antigen structure, though if a reliable template is available it may be possible to start with a homology model generated computationally, for example, using tools, such as I-Tasser or Rosetta, which have performed strongly during recent CASP tests [93]. We anticipate that these proofs of principle will pave the way for similar epitope grafting strategies targeting the variable antigens of alternative pathogens where strain variation causes incomplete protection upon immunization with antigens from one strain only.

## 5. Enhancing Antigen Homogeneity and Stability

A second area where structural and computational biology have been combined synergistically is in the rational optimization of an antigen that presented the confounding issue of conformational heterogeneity. This approach was illustrated by research performed to identify an effective vaccine antigen to protect against respiratory syncytial virus (RSV). RSV is an important unmet medical need; it is the main viral cause of severe respiratory tract disease in children worldwide, being responsible for approximately 6% of infant deaths [118, 119] and also affecting immunocompromised adults and the elderly [120]. The fusion glycoprotein F is an obvious candidate vaccine antigen, and indeed is the target of a licensed therapeutic mAb (palivizumab, or Synagis) [121]. The F protein is a well-conserved trimeric surface antigen of 150 kDa, but vaccine development was hampered by its conformational variability, typical of viral fusion glycoproteins that undergo large structural changes from prefusion to postfusion states when mediating membrane fusion [122]. In short, while prefusion F might conceptually be the preferable antigen due to its exposure on the virion, it is only metastable as a recombinant protein and converts into a postfusion conformation. In contrast, a simple postfusion antigen was unsuitable, due to its tendency to aggregate, caused by an exposed hydrophobic fusion peptide segment [123].

A promising postfusion F candidate antigen for RSV was rationally designed by removal of the hydrophobic fusion peptide, the transmembrane segment and the cytosolic region. The resulting antigen was readily produced, nonaggregating, homogeneous, highly thermostable and presented the key neutralizing epitopes recognized by palivizumab. Moreover, this postfusion F construct raised high titers of neutralizing antibodies in rodent models of RSV infection, suggesting that it is a promising antigen for clinical trials to protect against RSV [124].

Subsequently, it was reported that much of the neutralizing activity present in human sera after infection targeted the prefusion F state [125]. Design of a stable prefusion F construct was not feasible based on previously known structures but became possible following cocrystallization and structure determination of F in complex with the prefusion-specific human Fab D25 [126]. This antibody-antigen complex provided a platform for the computer-assisted design of point mutations to stabilize the protein in the conformation captured by the antibody. Analysis of C*β*–C*β* bond distances enabled design of a number of Cys substitutions that would introduce disulfide bonds that might covalently lock the F protein in the prefusion conformation (with the most successful pair of mutations being S155C and S290C). In addition, structural analysis revealed a number of sites where amino acid substitutions could enhance protein stability by filling only partially occupied cavities and thus increase hydrophobic packing interactions, in particular the cavity-filling mutation S190F. The designed antigen was further stabilized in its trimeric state by a C-terminal foldon domain, added specifically to ensure stable trimerization. This novel prefusion F candidate was subsequently shown to induce high titers of neutralizing antibodies in rodent and nonhuman primate models [19]. Collectively, the various studies performed to generate both pre- and postfusion F antigen candidates demonstrate how structural studies, computational analyses, and modeling can guide site-directed mutagenesis to generate novel antigens for consideration in RSV vaccine trials.

The HIV-1 Env surface glycoprotein has also been a target for extensive research in the structural vaccinology field. Until recently, the structure of the native prefusion trimer had remained elusive. With the help of an engineered disulfide bond between the GP120 and GP41 subunits and an additional stabilizing mutation required to keep the GP41 in its prefusion conformation, a stable BG505 SOSIP.664 construct was obtained and crystallized and its structure was solved alone and in complex with bnAbs PGT122 and 35O22 [127–129]. Immunization of rabbits and macaques with SOSIP.664 constructs induced autologous neutralizing antibodies, a highly promising sign as the major hurdle in HIV vaccine development has been the inability to induce germline B-cells to mature and mutate to secrete the required bnAbs [130]. Recent breakthrough research indicates that a successful strategy for HIV immunization is likely to be composed of several temporally separate injections of which the first ones contain antigens capable of efficiently stimulating the rare B-cell precursors and subsequent injections containing native-like Env that stimulate the already activated B-cell populations to undergo further somatic mutation thus evolving to bind the native Env on the virion surface [131, 132]. Such a germline-targeting immunogen could be the minimal engineered outer domain (eOD) assembled on nanoparticles developed by Jardine et al. [132] and a native-like Env construct could be that recently developed through structure-based design on the SOSIP.664 background by Do Kwon et al. [20]. Structure-based optimization of a vaccine antigen has also been used for* Borrelia burgdorferi* outer surface protein A [133]. The authors used NMR epitope mapping to reveal that protective epitopes were located exclusively on a C-terminal globular domain. Based on this knowledge, a truncated version of the molecule was designed and produced but found to be unstable and to induce poorer protection in mice compared to the wild-type protein. Replacing some of the charged residues within the core of the domain by hydrophobic residues improved the stability of the construct to levels similar to the wild-type, and likely as a consequence of the increased stability, the construct was found to be equally good as the wild-type in eliciting protective immunity in mice.

## 6. Optimizing Epitope Presentation

Vaccinating with native antigens is not always optimal and engineered constructs containing only the protective epitopes may perform better in eliciting the optimal immune response. An important example is provided by the influenza haemagglutinin (HA) where immunization with the native protein in seasonal influenza vaccines drives a response mainly directed to the highly variable head region of this antigen resulting in the need to develop a new vaccine almost every year to fight the strains prevalent during a given year [134]. A growing body of evidence suggests that the much less variable stalk region of HA contains neutralizing epitopes, and is therefore a rational point of focus in developing HA antigens [134]. The challenge here is that, in order to direct the immune response to the stalk, the interfering effects of the variable immunodominant head have to be circumvented. The most common approach has been to attempt to make constructs containing only the stalk region. These constructs have had the tendency to be poorly producible in soluble form and to adopt the post-fusion conformation, where neutralizing epitopes are not retained. Recently, however constructs faithfully reproducing the pre-fusion stalk and capable of inducing bnAbs have been reported [135, 136]. Mallajosyula et al. used a computational minimalization approach to design fragment constructs that, basing on interaction network analysis, contained only the residues essential to faithfully reproduce the epitopes. Hydrophobic residues outside the epitope were mutated to prevent aggregation and the fragments connected by flexible linkers and trimerization enhanced by isoleuzine zippers or foldon domains. The obtained antigens were able to elicit bnAbs and confer robust protection against lethal, heterologous viral challenge in mice [136]. Another approach shown to improve the elicited antibody titers to HA, including bnAbs to the stalk, is to express the full-length protein on the surface of ferritin nanoparticles [137]. While none of the HA antigens reported so far can be considered as a universal influenza antigen, the promise is that through further design and engineering an antigen capable of inducing neutralizing antibodies to most if not all influenza strains can be developed.

The examples described above demonstrate how knowledge of an antigen structure and its protective epitopes can be combined to generate novel vaccine candidates with improved characteristics based on closely related predefined scaffolds. However, it is also conceivable to identify protective epitopes that can be targeted by neutralizing antibodies and mount them as conformationally relevant fragments on non-related scaffold structures, thus enabling new degrees of freedom in antigen design that may overcome issues related to intrinsically problematic behavior of the full-length antigen. For example, as described above, the native RSV F antigen displays properties unsuitable for development as a vaccine antigen (e.g., meta-stability, or tendency for aggregation). Therefore, alternative methods were sought to enable design of novel RSV F conformational epitope presentation strategies, since the known epitope did not elicit neutralizing antibodies when used as a peptide immunogen [138], likely to due to lack of appropriate conformation of the unconstrained peptide. Briefly, the neutralizing epitope targeted by the therapeutic mAb (palivizumab) was characterized by determination of its crystal structure in complex with the Motavizumab Fab—an affinity-enhanced derivative of palivizumab with a picomolar dissociation constant (*K*_*D*_) for the same epitope in full-length F [29]. The complex structure revealed a highly complementary paratope/epitope interface, with the epitope fragment of 24 residues in a helix-turn-helix conformation making many contacts between the two helices and the Fab. Initial attempts were made to computationally screen all known structures in the Protein Data Bank (PDB) that might be able to host this F-derived helix-turn-helix motif in a conformationally faithful manner, thus enabling epitope presentation on a heterologous scaffold. Indeed, a subset of structures was identified and subsequent grafting of the F epitope into three of these structures was attempted. One of these epitope scaffolds bound Motavizumab with reasonable kinetics (although with an affinity considerably lower than the native F protein). However, when tested in mice, although this immunogen did induce antibodies able to recognize the F antigen, the immune sera lacked RSV neutralizing activity [30]. There was not a clear explanation for the apparent inability to elicit a protective response, which may be linked to the lower affinity observed for Motavizumab binding or to insufficient epitope mimicry or because additional epitopes outside the helix-turn-turn motif are required.

**Figure 3:**
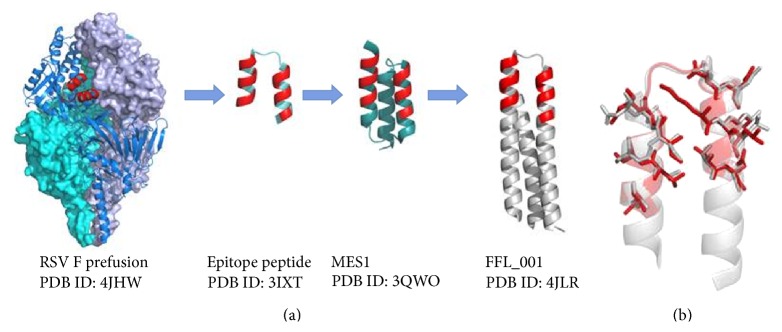
Development of a respiratory syncytial virus (RSV) F scaffold antigen. (a) RSV F Motavizumab epitope is conserved in both pre- and postfusion conformations and a peptide epitope in complex with Motavizumab was shown to have a similar conformation as the epitope in RSV F (PDB ID: 2IXT) [29]. This information was used to design scaffolds presenting the epitope of which the best, MES1 (PDB ID: 3QWO), bound Motavizumab with high affinity but failed to induce protection in mice upon immunization [30] FFL_001 was the first example of a computationally designed scaffold that when used in immunization of macaques induced protection against the virus (PDB ID: 4JLR) [31]. FFL_001 was obtained through a computational* de novo* epitope scaffold design approach called “Fold From Loops” aimed at faithfully reproducing the epitope with strong immunogenic properties. The epitope on RSV F and the residues responsible for interaction with Motavizumab on the antigen constructs are shown in red. (b) Overlay of the RSV F epitope from prefusion F and the epitope region from FFL_001 illustrates the faithful reproduction of the epitope in FFL_001. Epitope residues important for Motavizumab binding from RSV F are shown in red and the corresponding residues from FFL_001 in gray.

To further develop the epitope scaffold strategy, Correia et al. devised new computational methods to design* de novo* scaffold proteins more ideally suited to display and accurately mimic the RSV neutralizing F epitope [31]. Their method, termed “Fold From Loops (FFL),” has four main steps: (i) selection of the functional motif and target topology to host the motif, (ii)* ab initio* folding to identify suitable main chain structures, (iii) iterations to select the most compatible low-energy side chain solutions, and (iv) automated and human-guided fine tuning to select the best structural candidates. In the specific test case, the latter step involved manual replacement of surface-exposed residues outside the epitope with those from the scaffold template protein and the computational selection of large hydrophobic residues to be inserted within the buried protein core. Importantly, leading designs were biophysically and structurally characterized, and at least eight constructs displayed key signs of being soluble and monomeric, with correct folding and high thermostability (*T*_*m*_ > 75°C); several also bound to Motavizumab with high affinity (*K*_*D*_ 6–94 pm), suggesting their faithful reproduction of the neutralizing epitope on these scaffolds (*K*_*D*_ for wild-type F glycoprotein was 35 pm), confirmed by crystal structure determinations, and thus representing a major improvement on their previous efforts. Ultimately, the epitope scaffold designs were tested in mice and nonhuman primate animal models. Macaques produced robust binding responses against the autologous antigen scaffolds and RSV F protein, and neutralizing activity was detected in sera in up to 12 of 16 animals. Notably, some of the animals had neutralization titers comparable to those induced by natural human infection. We illustrate the development pathway from the F prefusion Motavizumab epitope to the latest protection-inducing scaffolds of Correia et al. in Figure 3. To summarize, this new structural and computational approach enabled generation of novel epitope scaffolds with robust antigenic properties and presented the F epitope in the desired conformation, as confirmed experimentally by structure determination alone or in complex with Fabs and by immune recognition using sera from RSV-seropositive humans. These studies, which included preclinical experiments in nonhuman primates, ultimately provided a proof of principle for the design of epitope-focusing scaffolds that can successfully elicit neutralizing antibodies against a desired protective epitope. Clearly, this approach could be applied to the design of antigens against a variety of pathogens and could potentially be further developed by the incorporation of multiple epitopes per scaffold, thus increasing breadth of protection elicited by the antigen. Indeed, in the search for potent antigens to protect against HIV, a few promising studies have been performed using scaffolds to stably display portions of gp120 or gp41 [139–141]. Ultimately, the structure-based computational design of epitope scaffolds appears to be a versatile and high-precision approach to vaccine discovery that holds great promise.

## 7. Conclusion

Structural and computational biology have become important in designing vaccines against diseases unamenable to traditional empirical vaccine development strategies. Computational antigen selection tools are now sophisticated enough to allow a relatively straightforward selection of a limited number of vaccine antigen candidates from whole genomes as a starting point for vaccine development.

However, bioinformatics predictions can fail to correctly identify the posttranslational modifications such as glycosylation, phosphorylation, and molecular rearrangement following proteolytic cleavage that can change the structure and potentially the antigenic properties of bacterial antigens. Integration with proteomics can represent a valid strategy to refine the antigen characterization as well as provide useful insights on abundance and subcellular localization of bacterial antigens [46, 142, 143].

With the increasing speed of X-ray crystallographic structure determination and the promise of cryo-EM in rapid high-resolution structure determination, the number of antigen-antibody complex structures in the PDB is expected to rise at an increasing speed. A significant contribution to this will likely be provided by the fast characterization and cloning of antibodies enabled by B-cell sequencing. The availability of more experimental structures will also help in developing more reliable computational tools for epitope prediction as well as in designing scaffolds for epitope presentation; that is, the more we know, the more we can predict.

Recent developments in epitope-oriented scaffold-based antigen design show great promise but still require additional successful examples to become the norm. A burning question, especially in the case of HIV, is that to what extent the epitope information of bnAbs is useful for vaccination purposes since the germline B-cell receptors that need to first recognize the antigen are very diverse from the bnAbs after somatic hypermutation.

We expect that structural optimization in terms of thermostability, conformational heterogeneity, and safety are likely to show up as the first examples of SV in the pool of vaccines for approved use in human. Indeed, it will be very interesting to see how many of the promising preclinical candidates perform in clinical trials and which will get the privilege to lead the way for other SV-based vaccines of the future.

## Figures and Tables

**Table 1:** Software for antigen discovery.

Software	Vaccine candidate prediction targets			Main selection criteria	Multipass membrane protein/human homolog detection	Host-pathogen interactions	MHC epitopes
	Prokaryotes	Archaea	Eukaryotes	Subcellular localization			
Jenner-Predict [32]	+	−	−	+	+	+	−
NERVE [33]	+	−	−	+^3^	+	+	−
PSORTb 3.0 [34]	+	+	−	+^2^	−	−	−
Vacceed [35]	−	−	+	+	+	−	+
Vaxign [36]	+	−	−	+^2^	+	+	+
VaxiJen v2.0^1^ [37]	+	−	−	−	−	−	−

^1^VaxiJen uses an alignment-independent approach making it not directly comparable to the other software in the antigen selection criteria.

^2^Using PSORTb 2.0 for subcellular localization prediction.

^3^Using PSORTb 3.0 for subcellular localization prediction.
